# Regular Dilatation in Tubularized-Incised Urethral Plate Urethroplasty for Prevention of Fistula Formation

**DOI:** 10.5812/ircmj.6871

**Published:** 2013-12-05

**Authors:** Mohammad Zarenezhad, Seyed Mohammad Vahid Hosseini

**Affiliations:** 1 Legal Medicine Research Center, Legal Medicine Organization, Tehran, IR Iran; 2 Department of pediatric surgery, Shiraz and Hormozgan University of Medical Sciences, Shiraz, IR Iran

**Keywords:** Urethra, Fistula, Primary Prevention

**Dear Editor,**

Tubularization of an entirely incised urethral plate is reported to have satisfactory functional and cosmetic results even in proximal hypospadias (1). In this technique, the urethral plate is divided into two epithelial strips that are approximated in the midline ventrally however; the roof and the side-walls are formed by raw surface that will eventually be covered by epithelium of plate regenerating (2). The stenosis of the neomeatus was associated with post-operative fistula (1).

In our experience, thirty five patients (mean age 2.5 years, range 2–10 years old), whose hypospadias were corrected using the tubularized-incised urethral plate technique, were followed-up. Ten had coronal, and twenty five had mid shaft hypospadias. The urethral plate was incised in the midline and tubularized over a proper stent. A local anaesthetic gel containing lidocaine hydrochloride was applied and the neourethra gently dilated using a Nelaton catheter of the same size as the intra-operative stent. This manoeuvre prevented adhesions between the raw surfaces of the incised urethral plate. Of total 35 patients, ten patients had a small fistula associated with meatal stenosis. By regular dilatation of the urethra, six fistulae resolved spontaneously and the remaining patients cured surgically. Other complications included hematoma formation in five patients and urinary tract infection in two patients.

Jordan and Schlossberg (3) explained the cause of post-operative meatal stenosis as epithelial apposition is prevented by separating both sides of the wound, healing occurs by secondary intention and the epithelium progresses slowly from the edges of the wound to cover the raw surface. The natural tendency of wounds to contract will aid epithelial apposition; limits the size of the area requiring epithelialization and promotes stenosis that make the urethroplasty developed fistula.

The most reliable method to counteract the process of urethral wound contraction is long-term and regular dilatation of the urethra (4-6). Neourethral dilatation is easy, relatively painless and can be administered at home by parents for 1–2 weeks. Older children, adolescents and young adults were able to perform daily dilatation themselves with no problems. Regular urethral dilatation is important in preventing adhesions between both sides of the incised plate, which can result in meatal stenosis and fistula.
